# Better overall survival in patients who achieve pathological complete response after neoadjuvant chemotherapy for breast cancer in a Chilean public hospital

**DOI:** 10.3332/ecancer.2021.1185

**Published:** 2021-02-11

**Authors:** Francisco Acevedo, Militza Petric, Benjamin Walbaum, Julieta Robin, Luisa Legorburu, Geraldine Murature, Constanza Guerra, Marisel Navarro, María José Canovas, Cesar Sanchez, Lorena Vargas, Manuel Manzor, José Peña, Sabrina Muñiz, Paulina Veglia, Raúl Cartes, Raúl Martinez

**Affiliations:** 1 Haematology-Oncology Department, Pontifical Catholic University of Chile, Diagonal Paraguay 319, Santiago 8330032, Chile; 2 Dr Sotero del Rio Health Complex, Av Concha y Toro 3459, Puente Alto, Santiago 8207257, Chile; 3 Chilesincancer Foundation, Isidora Goyenechea 3477, 5 piso, Las Condes, Santiago 7550106, Chile; 4 Dr Eloisa Diaz Clinical Hospital, Froilan Roa 6542, La Florida, Santiago 8242238, Chile; 5 Padre Hurtado Hospital, Esperanza 2150, San Ramon, Santiago 8880465, Chile; 6 Iram Clinic, Americo Vespucio Norte 1314, Vitacura, Santiago 7630370, Chile

**Keywords:** breast cancer, neoadjuvant therapy, prognosis, public hospital

## Abstract

**Introduction:**

There is extensive evidence associating the response to neoadjuvant chemotherapy (NeoCT) with breast cancer (BC) survival. However, to the author’s knowledge, there is no published data in Chile. The objective of the study is to evaluate whether achieving pathological complete response (pCR) after NeoCT is associated with greater survival and lower risk of recurrence in a Chilean Public Health Service.

**Methods:**

Retrospective analysis of a database. Patients with a diagnosis of Stages I–III BC who received NeoCT between 2009 and 2019 were included. Clinical and pathological information were extracted from the clinical records. BC subtypes were defined using hormone receptor (HR) information (HR: oestrogen and/or progesterone) and epidermal growth factor type 2 (HER2), being divided into four groups: HR+/HER2−, HR+/HER2+, HR−/HER2+, HR-/HER2−. pCR was defined as the absence of invasive cancer in the breast and axilla (ypT0/is N0) after NeoCT.

**Results:**

Of 3,092 patients, 17.2% received NeoCT. Of these, 40.2% corresponded to HR+/HER2−, 20.9% HR+/HER2+, 18.2% HR−/HER2+ and 20.7% HR−/HER2−. Overall, 24.8% achieved pCR, being the lowest for HR+/HER2− (10.3%) and the highest for HR−/HER2+ (53.2%). In the multivariable analysis, family history, HER2+ and type of chemotherapy were associated with a greater probability of pCR. With a median follow-up of 40 months, the overall survival and metastasis-free survival (MFS) at 3 years were greater for the group with pCR compared to that which did not achieve it (90.5% versus 76.7%, *p* = 0.03 and 88.5% versus 71.4%, *p* = 0.003, respectively). The multivariable analysis confirmed this finding. Brain MFS was similar in both groups.

**Conclusion:**

NeoCT is associated with greater pCR in aggressive BC subtypes. In those, achieving pCR was associated with better survival in our study. To the author’s knowledge, this is the first study which evaluates the relation between pCR and BC subtypes in a Chilean public hospital.

## Introduction

Breast cancer (BC) is the leading cause of death from neoplasms in Chilean women [1]. It is characterised by being a highly heterogeneous disease demonstrated by diverse biological behaviour and highly varied probability of responding to different therapies. Based on the expression of genetic profiles, molecular subtypes have been identified in BC [2]. The luminal subtypes are characterised by the expression of genes related to oestrogen receptors (ER). Moreover, these can be subclassified according to low (Luminal A) or high (Luminal B) expression of genes related to cellular proliferation such as epidermal growth factor type 2 (HER2) or Ki-67. HER2-enriched subtype is characterised by high proliferation of genes related to HER2 and low expression of ER, and the basaloid subtype which is characterised by low expression of genes associated with HER2 and ER, but express genes such as Epidermal Growth Factor Receptor (EGFR), cytokeratins 5/6 and c-kit [3]. One of the advantages of this genetic classification is that it has a phenotypical correlation with classical immunohistochemical (IHC) tests using HRs such as ER or progesterone receptor (PR), HER2 and Ki-67 according to those shown in Table 1.

All of those tumours which show over-expression of HER2 or which do not express HR or HER2 (also called Triple Negative (TN)) are characterised by a worse prognosis with a high risk of recurrence, metastasis and mortality [4].

Neoadjuvant therapy refers to the use of some treatment, normally chemotherapy (CT), prior to surgery with curative intention. Even though pivotal studies, such as the National Surgical Adjuvant Breast and Bowel Project (NSABP) B-18 and B27 [5], demonstrated that the benefit of NeoCT is to reduce the volume of a tumour and so perform less invasive surgeries; these data did not translate into a survival benefit on comparing this approximation with post-surgical treatment. This meant that for a long time NeoCT was used only in those patients with locally very advanced tumours or in those for whom it was desirable to preserve the breast. However, two facts led to NeoCT becoming the standard treatment for many patients, regardless of the surgery to be performed.

The first was its prognostic value. Studies subsequent to that of the previously mentioned NSABP showed that for the tumour to disappear completely, both in the breast and the axilla, which would be defined as Pathological Complete Response (pCR), is associated with less risk of recurrence and greater survival [6]. This fact is much clearer in those aggressive subtypes (TN or tumours which over-express HER2) [7, 8]. Such has been the impact of this association that the Food and Drug Administration for the first time gave a conditional approval of a drug based only on the use of the medication in this parameter [9].

The second is its predictive value. Patients with HER2+ tumours who do not achieve pCR are candidates to receive trastuzumab-emtansine in place of trastuzumab after surgery given its benefit in recurrence [10]. Those patients with TN tumours who also do not achieve pCR should be considered for the use of capecitabine after surgery because of its benefit in overall survival (OS) [11].

The aim of this study is to evaluate pCR in the Chilean population and to analyse if it is associated with subtypes and survival.

## Methods

### Patient selection

Retrospective analysis of a database of patients treated for invasive BC in the South Eastern Metropolitan Health Service (Servicio de Salud Metropolitano Suroriente—SSMSO) in Santiago, Chile. This service includes the following public hospitals: Sotero del Rio Hospital (HSR), La Florida Hospital (HLF) and Padre Hurtado Hospital (HPH). The study was approved by the local ethics committee. All those patients were included who had a diagnosis of Stages I–III BC and who were diagnosed and treated with NeoCT between January 2009 and December 2019. Patients were excluded who had metastases on diagnosis, those who received neoadjuvant hormonal treatment as an exclusive treatment and those for whom we did not obtain information about the pathological response in the surgical biopsy.

### Data collection and categorisation

The clinical, pathological and follow-up information were extracted from the electronic clinical record. Survival data were extracted from the civil registry. Given that: (1) we do not have Ki67 data for all the patients and (2) for those we have, there are doubts about the correct standardisation of this technique across the different laboratories [12]; it has been decided to use only information about HR and HER2 to divide the tumours. The cut-off value for ER and/or PR was >1% and HER-2 was considered positive if in the IHC it was 3 pluses or Fluorescent *In Situ* Hybridization (FISH) positive it was 2 pluses. In this way, we obtained four groups: HR+/HER2− (luminal A or B, HER2−), HR+/HER2+ (luminal B-HER2+), HR−/HER2+ (HER-2 enriched) and HR−/HER2− (TN).

### Pathological complete response

The pCR was evaluated, which was defined as the absence of invasive cancer, both in the breast and in the axilla (ypT0/is N0) after NeoCT.

### Statistics

Descriptive statistics were used to describe basal characteristics using Chi-squared to compare categorical variables and Kolmogorov–Smirnov for continuous variables. A uni and multivariable logistical regression were used to evaluate the association of clinical and pathological variables with pCR. For the multivariable analysis, only those factors which showed significance in the univariate were considered. Metastasis-free survival (MFS), Cerebral MFS and OS were calculated from the date of diagnosis to the first event, or loss of follow-up and were presented in Kaplan–Meier curves. The groups were compared using log-rank. A univariate and multivariable Cox or logistic regression was carried out to evaluate factors associated with survival. Once more, for the multivariable analysis, only those factors which gave significance in the univariate regression were considered. A *p* < 0.05 was considered to define statistical significance. STATA 15.1 software was used for all the analyses.

## Results

**Figure 1.**
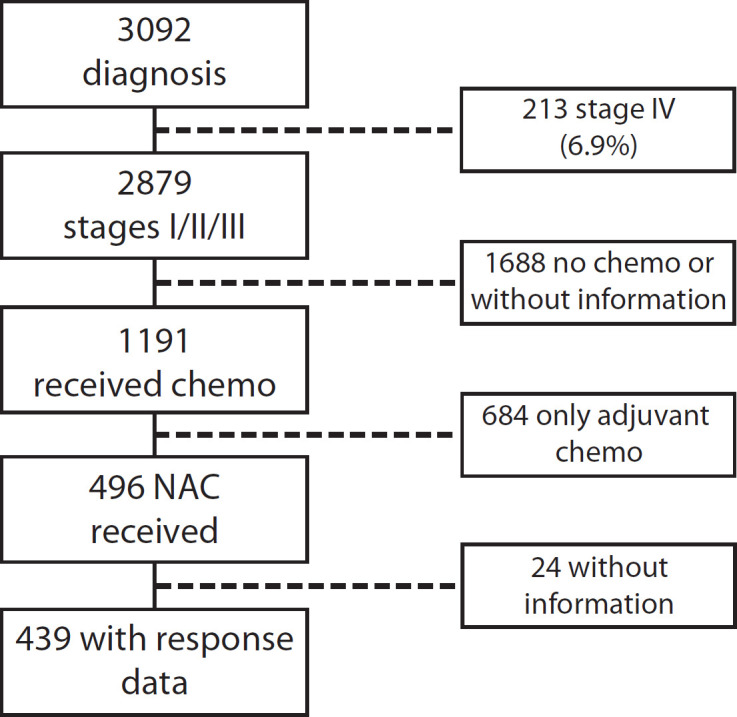
Patient flowchart.

**Figure 2.**
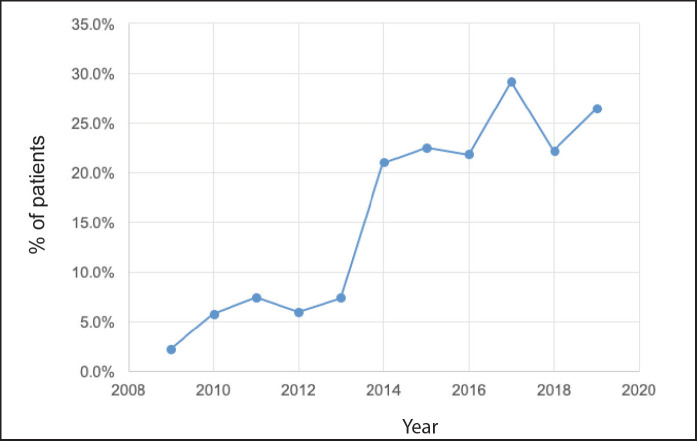
Proportion of patients who receive NeoCT per year considering all patients diagnosed in Stages I–III.

Of a total of 3,092 patients diagnosed between 2009 and 2019, 2,879 corresponded to Stages I–III (93.1%) patients. Of these, 496 (17.2%) received NeoCT (Figure 1). The proportion of Stages I–III patients who received NeoCT varied according to the year of diagnosis. There was an increase from 2.2% in 2009 to 26.5% in 2019 (Figure 2). Of the 496 patients analysed, information about the type of response achieved was obtained in 439, which were included in this study. The characteristics of these patients are provided in Table 2.

The median age was 51.7 years (range = 23–79 years). Of these, 40.3% (*n* = 176) were HR+/HER2−, 20.8% (*n* = 91) HR+/HER2+, 18.1% (*n* = 79) HR−/HER2+ and (*n* = 91) 20.8% HR−/HER2−. The majority of patients (58.3%, *n* = 240) corresponded to clinical Stage III.

**Figure 3.**
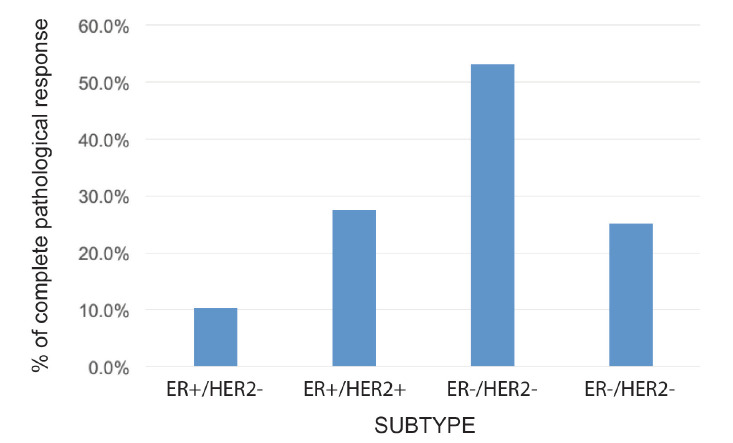
Rate of pCR according to receptor status.

The rate of pCR in the whole group was 24.8% (109 patients). This rate varied according to subtype: HR+/HER2− 10.3%; HR+/HER2+ 27.5%; HR−/HER2+ 53.2%; and HR-/HER2− 25.1% (*p* = 0.0001) (Figure 3). On subdividing the subgroup HR+/HER2− into Luminal A (LA) or Luminal B (LB) according to histological grade (HG) and/or Ki-67, the pCR was 0.0% versus 21.0%, respectively (*p* = 0.001).

56.5% of the patients had a family history (FHx) of any cancer, being more frequent in patients who achieved pCR than those who did not (*p* = 0.0001). There were no significant differences on evaluating the FHx of BC (Table 2). 57% of the patients met indications for genetic counselling according to National Comprehensive Cancer Network (NCCN) guidelines [13]. Genetic studies were only carried out on eight patients, and six of those had a mutation on *BRCA* 1 or 2 genes.

**Figure 4.**
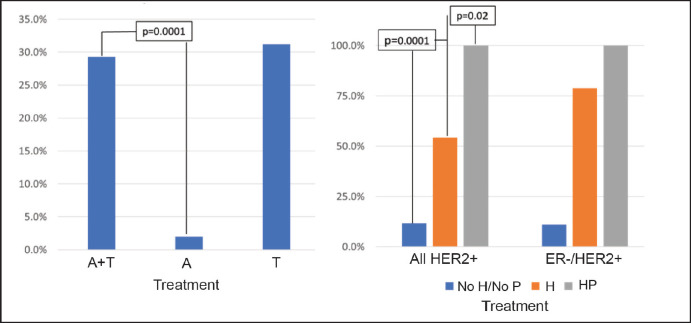
Rate of pCR according to treatment received. (a): pCR according to CT used. (b): pCR in HER2+ patients according to anti-HER2 treatment received. A = anthracyclines only, A-T = anthracyclines and taxanes, T = taxanes only, H = trastuzumab, P = pertuzumab.

The majority of the patients (84.4%) received anthracyclines and taxanes (A-T) as a NeoCT regime, with 19.0% using a dense dose of these. On considering only those patients using the A-T CT scheme, the pCR rate was 29.3% (Figure 4a) and this varied according to subtype: HR+/HER2− 12.0%; HR+/HER2+ 32.0%; HR−/HER2+ 66.1%; and HR−/HER2− 30.3% (*p* = 0.0001). There was no significant difference on analysing the density of the dose or use of platinum. In HER2+ patients, 75.9% received trastuzumab as a neoadjuvant treatment, and six of them (4.3%) also received pertuzumab (two in the HR+/HER2− group and four in the HR−/HER2− group). The use of neoadjuvant trastuzumab was associated with a greater rate of pCR (54.2% versus 11.8%, *p* = 0.0001), making this difference more marked for HR−/HER2+ patients (78.7% versus 11.1%, *p* = 0.0001) (Figure 4b). All the patients who received trastuzumab combined with pertuzumab achieved pCR (*p* = 0.02 compared with trastuzumab without pertuzumab).Regarding the type of surgery received after NeoCT, 56.3% had a total mastectomy (TM) being less frequent in patients who achieved pCR versus than those who did not achieve it (45.2% versus 59.9%, *p* = 0.01). The rate of axillar dissection was 85% in both groups (*p* = 0.87).

**Figure 5.**
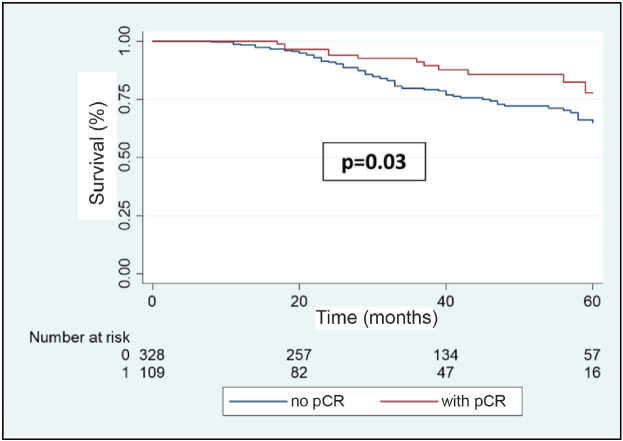
OS in patients with BC after NeoCT according to pCR versus no-pCR.

**Figure 6.**
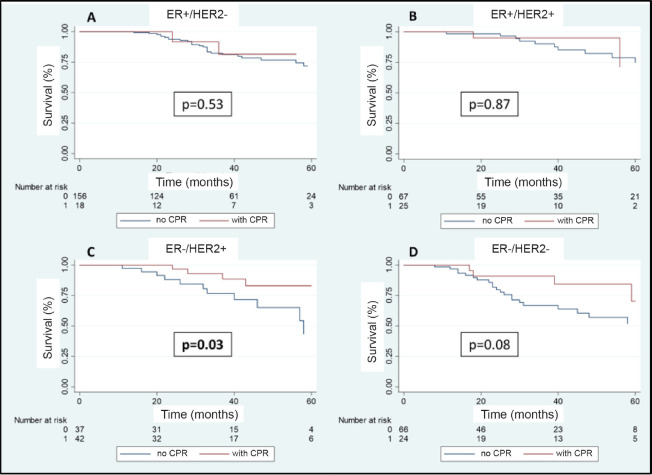
(a–d): OS in patients who achieve pCR versus no-PCR according to receptor status.

In the multivariable analysis, considering only those factors which were significant in the univariate analysis (Table 3), both the positivity of HER2 (odds ratio 5.3) and the FHx (OR 2.3) were associated with pCR. Moreover, the presence of HRs in the tumour reduces by 70% the probability of achieving pCR independent of the HER2 status or the FHx. On the other hand, the use of regimes with anthracyclines associated with taxanes increases the chance of pCR more than 13 times in comparison to regimes which only use anthracyclines, independently of receptors or FHx. With an average follow-up of 40 months, a total of 94 deaths were observed, 81 of these in patients who did not achieve pCR and 13 in patients with pCR. The OS at 3 years was 81.7% for the whole group. The OS at 3 years in the group which achieved pCR compared with those who did not achieve it was 90.9% versus 79.5% (*p* = 0.03) (Figure 5). The OS at 3 years according to subtypes and achieving pCR or not was the following: HR+/HER2− 82.1% versus 81.5% (*p* = 0.53); HR+/HER2+ 94.7% versus 90.1% (*p* = 0.87); HR−/HER2+ 92.9% versus 76.7% (*p* = 0.03); and HR−/HER2− 90.8% versus 66.6% (*p* = 0.08) (Figure 6a–d).

**Figure 7.**
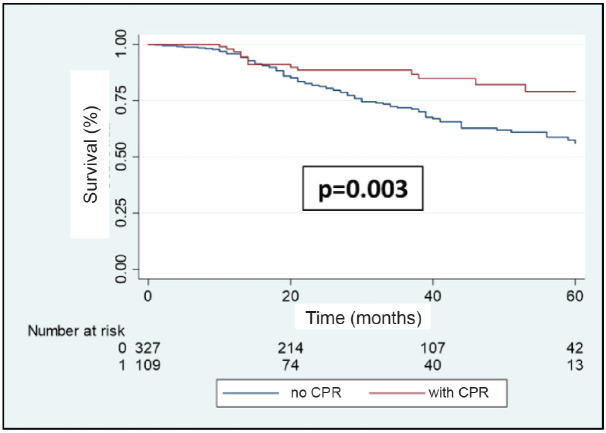
Metastases recurrence-free survival in patients with BC according to whether or not they achieve pCR after NeoCT.

**Figure 8.**
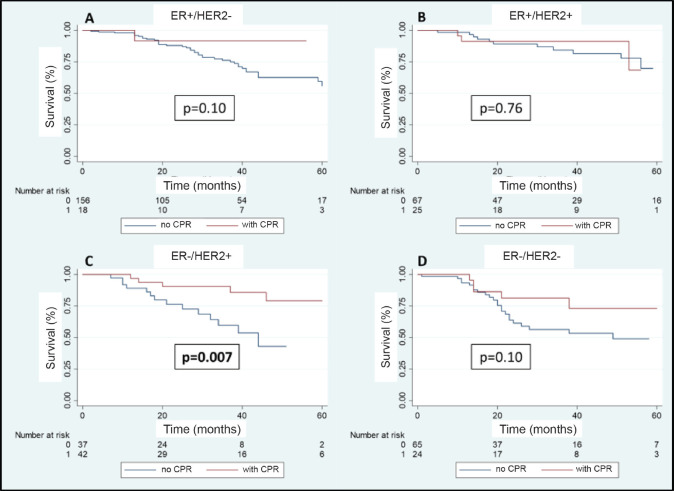
(a–d): Metastases recurrence-free survival in patients who achieve pCR versus no pCR according to receptor status.

A total of 108 patients were observed with distant recurrence, of these women 87.0% (*n* = 94) were patients who did not achieve pCR and 13.0% (*n* =14) of these had achieved pCR. The MFS at 3 years was 75.5% for the entire group. The MFS at 3 years for the group which achieved pCR compared to those who did not achieve it was 88.5% versus 71.4% (*p* = 0.003) (Figure 7). The MFS at 3 years according to subtypes and whether pCR was achieved or not was the following: HR+/HER2− 91.7% versus 75.8% (*p* = 0.10); HR+/HER2+ 91.2% versus 84.2% (*p* = 0.76); HR−/HER2+ 90.5% versus 59.1% (*p* = 0.007); and HR−/HER2− 81.3% versus 56.0% (*p* = 0.10) (Figure 8a–d).

**Figure 9.**
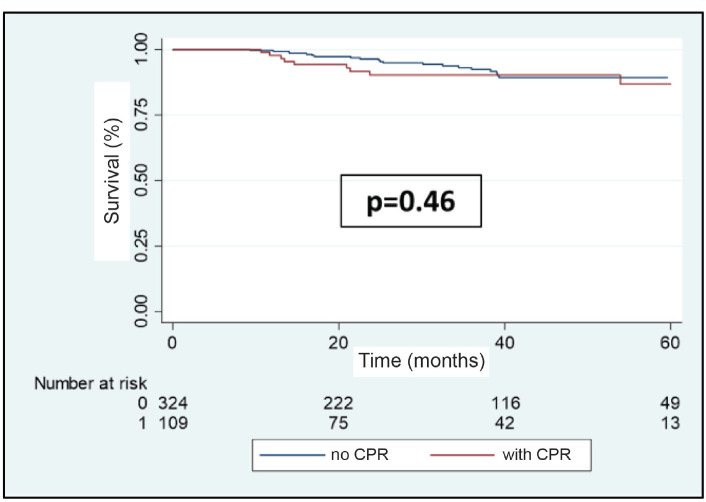
Cerebral metastases recurrence-free survival in patients with BC who receive NeoCT according to achieving pCR or not.

Of the 108 metastatic recurrences observed, 30 (27.8%) were in the central nervous system (CNS), either as cerebral and/or leptomeningeal metastases. Of these, 9 (30%) presented in patients who had achieved pCR, this being for 8 of these the only place where there was metastatic compromise. The brain metastases recurrence-free survival at 3 years was 90.4% for the group which achieved pCR versus 92.4% for the group which did not achieve it (*p* = 0.46, Figure 9).

Regarding the results of the multivariable Cox regression analysis (Table 4), our data show that the patients in Stage III at diagnosis have a risk of death from any cause and on presenting distant metastasis which is three times greater compared with patients in Stages I/II. Likewise, TN tumours are associated with a risk of death and distant disease 1.7 times greater compared with patients who have ER+/HER2− tumours. Moreover, compared with anthracyclines only, the combined use of anthracyclines and taxanes is associated with a better prognosis. To achieve pCR after NeoCT reduces the risk of death from any cause by 31% and the risk of distant metastases by 67%, whilst keeping the stage, subtype and treatment received constant.

## Discussion

To the author’s knowledge, this is the first study in the Chilean population which suggests that pCR subsequent to NeoCT is associated with better OS and lower probability of developing distant metastasis. Here, as is reported in international literature, the most aggressive subtypes (TN and HER2+) are associated with a greater probability of pCR [8].

NeoCT in BC is a treatment strategy which presents multiple benefits: first, on achieving a response and reducing the tumour load, it means that tumours which before were inoperable, become ‘operable’, or tumours which were initially going to be a TM can opt for more conservative surgeries. This benefit is the most obvious and has been reported in multiple studies [5]. In this study, the result was not different since patients who achieved pCR had TM rates significantly lower than those who did not achieve it, despite the lack of differences in clinical stage on diagnosis (Table 2). Although, there were no differences in the axillary dissection rate, which was notably high in both groups (85%), it is demonstrated that NeoCT managed to reduce this rate [14]. This can be explained by different reasons: (1) the lack of availability of the mixed media for the sentinel lymph node biopsy (SLN biopsy) post NeoCT throughout the hospitals of the network, opting instead to carry out SLN biopsy pre NeoCT only when patent blue is available; (2) that the practices of marking with a clip the positive biopsied lymph nodes (LN) has been recently incorporated, and (3) the high proportion of patients with locally advanced cancer T3/T4 and N2/N3 where ‘axillary downstaging’ would not be indicated.

But there are other benefits to NeoCT which, although they might be less obvious, they can have more of an impact. As already mentioned, achieving pCR could predict survival in patients with BC, especially in aggressive subtypes [8]. The other benefit is that it enables a ‘*live’* evaluation of the efficacy of different treatments. Therefore, patients who do not achieve pCR and hence have a greater probability of recurrence or dying from BC are considered resistant to the initial therapy, being a candidate to receive adjuvant treatments which overcome that resistance, thereby improving the oncologic prognosis in resistant patients, a finding which has been demonstrated in Phase III studies and incorporated into international management guidelines [10, 11, 15]. All these benefits have meant that NeoCT should be considered as a treatment option in patients with aggressive subtypes regardless of clinical status on diagnosis [16].For these reasons, this strategy has been adopted more and more frequently in developed countries. Mougalian *et al*. describe that in the United States, the rate of NeoCT in Stages I–III has doubled in the period 2003–11 from 12.2% to 24.0% [17], a fact that has also occurred in other European countries or Australia, although with smaller magnitude [18, 19]. This puts us in a good place within the international context. Since 2013, all the member hospitals of SSMSO proposed as an objective to increase the number of patients who should have NeoCT, an effort which is demonstrated in Figure 2, since then, achieving NeoCT rates over 20%.

However, not only is the intention to administer NeoCT important but ‘how we do it’ is also relevant. Regimes using anthracyclines combined with taxanes achieve a better rate of pCR (Figure 4) especially if compared with regimes which only use anthracyclines, treatment, we believe, should not be considered as a first alternative. In case that a patient should not be a candidate or does not want to receive anthracyclines, the use of regimes using taxanes like taxotere-cyclophosphamide is a valid option, with comparable pCR results as shown in Figure 4. Although the use of platinum and dense dose CT does not seem to significantly increase the rate of pCR, this result is limited by the number of patients exposed to this regime in our cohort. It would be interesting to study in the future in view of the randomised studies described in the literature [20–22]. On the other hand, it has been described that patients who have the germline *BRCA* mutation have a greater probability of achieving pCR particularly if platinum based regimes are used [23, 24]. In our case, this assessment is restricted by the limited number of patients we detected with this mutation. Although more than 50% of the patients fulfilled clinical criteria to be referred to genetic counselling, only 8 of these were studied (4.0%) managing to detect a genetic mutation in 6 of these (75%). One thing which it could suggest to us, if the presence of a mutation increases response, is the fact that patients who achieve pCR presented with greater probability of having a FHx than those without pCR (Table 2), this being the only associated factor, together with the BC subgroup for greater probability for pCR.

In 2016, a High Risk Hereditary Breast Cancer Programme was implemented in our centre. However, this high cost of the studies limited the access of those patients evaluated to carry out the genetic study. Since October 2019, the studies have been financed by the Hospital, which improved patient and family access. If the patient has indication to carry out the study according to NCCN clinical guidelines [13], the ideal is to carry them out during NeoCT, enabling a change in surgical treatment strategy if the result is positive and to consider interventions to reduce the risk of secondary primaries.

With respect to HER2+ patients, the NOAH (NeOAdjuvant Herceptin) study was the first randomised study to demonstrate the benefit of adding neoadjuvant trastuzumab, not only for achieving pCR but also for event-free survival [25]. Two subsequent randomised studies showed that the use of another anti-HER2 therapy such as pertuzumab, combined with trastuzumab, is associated with a better rate of pCR [26, 27]. Currently, the use of trastuzumab combined with pertuzumab and CT is an alternative treatment for HER2+ patients. This data is concordant with the results of our study. The rate of pCR increased significantly on adding trastuzumab to the normal CT regime, and seems to increase even more on adding pertuzumab, despite the limited number of patients in which this last strategy was used (Figure 4b). In view of this, all patients with HER2+ disease should receive anti-HER2 associated NeoCT therapy, unless any contraindication should present.

Although achieving pCR improves survival, this is not a synonym for cure. In our study, 10% of patients who achieved this result were dead at 3 years, the cause of death in nearly all these patients (92.3%) being BC (data not shown). On wanting to evaluate which are the clinical factors which could impact survival in patients who achieve pCR, we find that there is a tendency, which is not significant, that the nodal status on diagnosis could have an influence on survival. In a sub-study by European Organization for Research and Treatment of Cancer (EORTC) 10994/BIG 1-00, it was found that the clinical status on diagnosis was the only factor associated with prognosis in patients who achieved pCR [28]. Furthermore, interestingly, we find that obesity may have a prognostic role in patients who achieve pCR. Although, this characteristic has been described as a prognostic factor in BC subsequent to NeoCT [29], it is difficult to draw conclusions from our data given the limited number of patients evaluated.

Although our study found that pCR is associated with a lower probability of developing distant metastasis, this is not true for cerebral metastasis. In fact, 64.3% of patients who obtained pCR in our study developed CNS metastasis. This translated to there being no difference in brain MFS between those who achieved and those who did not achieve pCR (Figure 9). The reason for this probably has to do with the low probability of a classic cytotoxic regime such as anthracyclines or taxanes, or a big molecule like trastuzumab, they manage to penetrate the blood brain barrier leaving the CNS like a sanctuary free from CT. Finally, our study presents certain limitations which should be mentioned and could affect the applicability of our conclusions. The majority of these are inherent to retrospective studies. First, the study includes three public hospitals in Chile; therefore, it is probable that our results are not a reflection of the situation on a national level. Second, in comparison with international clinical studies, our report includes a relatively low number of patients considering the high prevalence of BC. Finally, given that our study is undertaken over an extensive period of time (2009–2019), changes in clinical guidelines and NeoCT regimes used should be borne in mind, which could themselves have affected the survival rates.

## Conclusion

In summary, achieving pCR subsequent to NeoCT seems to be a consistent and robust survival marker in patients with BC, in women treated in a Chilean public hospital. This prognostic value, together with the recently accepted predictive value of this marker, means that NeoCT should be considered as a standard treatment, particularly in patients with aggressive BC, regardless of clinical status.

## Conflicts of interest

The authors have no conflicts of interest to report.

## Funding statement

The authors have no funding to declare for this work.

## Figures and Tables

**Table 1.** Pathological correlation of intrinsic subtypes in BC.

Molecular or intrinsic subtype	IHC expression
Luminal A	ER+, PR+, HER2−, Histological Grade (HG) 1-2, Ki-67 low
Luminal B	ER+, PR+/−, HER2+/−, HG 2–3, Ki-67 elevated
HER-2 enriched	ER−, PR−, HER2+
TN	ER−, PR−, HER2−

**Table 2.** Patient characteristics.

	All patients	With pCR	Without pCR	*p*
Average age on Dx < 40 years	51.7 (23–79) 16.2%	48.2 (24–78) 14.7%	52.0 (23–79) 16.8%	0.33 0.61
Average BMI BMI > 30	28.2 (18.5–47.6) 31.0%	27.2 (18.5–44.7) 32.1%	28.5 (20.0–47.6) 30.5%	0.30 0.82
Clinical stage (TNM) I II III	2.0% 39.7% 58.3%	1.0% 39.6% 59.4%	2.3% 39.7% 58.0%	0.71
LN compromised No Yes	19.2% 80.8%	20.2% 79.8%	18.4% 81.6%	0.2
Average Ki67	40%	40%	30%	0.08
Subgroup by receptors HR+/HER2−HR+/HER2+ HR−/HER2+ HR−/HER2−	40.2% 20.9% 18.2% 20.7%	16.5% 23.0% 38.5% 22.0%	48.2% 20.3% 11.3% 20.2%	0.0001^a^
FHx of cancer FHx of BC First degree FHx of BC Fulfils NCCN guidelines for genetic counselling *BRCA* mutation	56.5% 30.3% 12.7% 57.0% 6 patients	74.1% 35.8% 12.3% 63.3% 1 patient	50.4% 28.4% 12.8% 54.9% 5 patients	0.0001^a^ 0.21 0.92 0.12 -
NeoCT treatment **Regime** Anthracyclines only Taxanes only Anthracyclines and taxanes (AC-T) **If AC-T** Dense dose With platinum **If HER-2+** Neo trastuzumab + Neo pertuzumab	11.8% 3.8% 84.4% 19.0% 3.1% 75.9% 4.3%	0.9% 4.6% 94.5% 20.4% 1.9% 93.5% 9.7%	15.6% 80.8% 3.6% 18.5% 3.6% 62.0% 0.0%	0.0001^a^ 0.67 0.41 0.0001^a^ 0.005^a^
Type of surgery TM Axillar dissection	56.3% 85.0%	45.2% 85.6%	59.9% 84.9%	0.01^a^ 0.87

a Statistical significance pCR: Pathological complete response, Dx: diagnosis, LN: lymph nodes, BMI: body mass index (weight/height^2^), HFx: family history, BC: breast cancer

**Table 3.** Univariate and multivariable logistical regression evaluating factors associated with pCR.

	Univariate analysis			Multivariable analysis^a^		
	OR	CI 95%	*p*	OR	IC 95%	*p*
Age at Dx	1.0	0.98–1.02	0.76	-	-	-
BMI	0.98	0.93–1.05	0.62	-	-	-
Clinical stage (TNM) I–II III	- 1.05	- 0.66–1.66	- 0.84	-	-	-
Compromised LN (yes)	0.88	0.51–1.54	0.66	-	-	-
Ki67	1.01	0.99–1.02	0.10	-	-	-
**HR positive**	**0.30**	**0.19–0.48**	**<0.001**^b^	**0.31**	**0.17–0.57**	**<0.001**^b^
**HER2 positive**	**3.50**	**2.22–5.47**	**<0.001**^b^	**5.26**	**2.92–9.50**	**<0.001**^b^
Subgroup by receptors HR+/HER2−HR+/HER2+ HR−/HER2+ HR−/HER2−	- 3.32 9.96 3.14	- 1.70–6.50 5.16–19.24 1.60–6.17	- <0.001^b^ <0.001^b^ 0.001^b^	- - - -	- - - -	- - - -
**FHx of cancer**	**2.81**	**1.61–4.91**	**<0.001^b^**	**2.31**	**1.23–4.35**	**0.009^b^**
NeoCT regime Anthracyclines only Taxanes only **Anthracyclines and Taxanes**	- 21.8 **19.8**	- 2.31–205.9 **2.70–145.8**	- 0.007^b^**0.003**^b^	- 7.82 **13.2**	- 0.48–127.6 **1.65–104.7**	- 0.15 **0.01^b^**

a In the multivariate analysis, only those factors were considered which were significant in the first analysis

b Statistical significance (*p* < 0.05)Dx: Diagnosis, LN: lymph nodes, BMI: body mass index (weight/height^2^), HFx: family history, BC: breast cancer, HR: hormone receptor

**Table 4.** Cox uni and multivariable logistic regression to evaluate factors associated with OS and metastases recurrence-free survival.

	OS				Metastases-free survival			
	Univariate		Multivariable		Univariate		Multivariable	
	HR	*p*	HR	*p*	HR	*p*	HR	*p*
Age at Dx	1.01	0.13	-	-	0.99	0.45	-	-
BMI	1.05	0.06	-	-	1.03	0.21	-	-
Clinical Stage (TNM) I–II **III**	- **3.55**	- **<0.001^a^**	- **3.05**	- **0.001^a^**	**-** **3.18**	**-** **<0.001^a^**	**-** **2.82**	**-** **<0.0001^a^**
Compromised LN (yes)	5.4	0.001^a^	-	-	2.80	0.003^a^	-	-
**pCR**	**0.52**	**0.03^a^**	**0.69**	**0.009^a^**	**0.43**	**0.003^a^**	**0.48**	**0.02^a^**
HR positive	0.62	0.02^a^	-	-	0.62	0.01^a^	-	-
HER2 positive	0.78	0.26	-	-	0.70	0.08	-	-
Subgroup by receptors HR+/HER2− HR+/HER2+ HR−/HER2+ **HR**−**/HER2**−	- 0.81 1.17 **1.73**	- 0.49 0.61 **0.03^a^**	- 1.40 1.34 **2.56**	- 0.33 0.42 **0.001^a^**	- 0.67 1.07 **1.68**	- 0.16 0.79 **0.03^a^**	- 1.04 1.60 **2.63**	0.89 0.14 **<0.001^a^**
FHx of cancer	0.72	0.18	-	-	0.70	0.11	-	-
NeoCT regime Anthracyclines only Taxanes only **Anthracyclines and Taxanes**	- 0.18 **0.38**	- 0.09 **<0.001^a^**	- 0.29 **0.55**	- 0.23 **0.03^a^**	- 0.28 **0.33**	- 0.09 **<0.001^a^**	- 0.57 **0.49**	- 0.45 **0.003^a^**
Adjuvant radiotherapy	0.57	0.09	-	-	0.63	0.17		
